# Meta-Analysis of Melanin-Concentrating Hormone Signaling-Deficient Mice on Behavioral and Metabolic Phenotypes

**DOI:** 10.1371/journal.pone.0099961

**Published:** 2014-06-12

**Authors:** Kenkichi Takase, Kenichi Kikuchi, Yousuke Tsuneoka, Satoko Oda, Masaru Kuroda, Hiromasa Funato

**Affiliations:** 1 Department of Anatomy, Toho University School of Medicine, Tokyo, Japan; 2 Department of Information Science, Faculty of Science, Toho University, Chiba, Japan; 3 International Institutes for Integrative Sleep Medicine, University of Tsukuba, Ibaraki, Japan; Kent State University, United States of America

## Abstract

The demand for meta-analyses in basic biomedical research has been increasing because the phenotyping of genetically modified mice does not always produce consistent results. Melanin-concentrating hormone (MCH) has been reported to be involved in a variety of behaviors that include feeding, body-weight regulation, anxiety, sleep, and reward behavior. However, the reported behavioral and metabolic characteristics of MCH signaling-deficient mice, such as MCH-deficient mice and MCH receptor 1 (MCHR1)-deficient mice, are not consistent with each other. In the present study, we performed a meta-analysis of the published data related to MCH-deficient and MCHR1-deficient mice to obtain robust conclusions about the role of MCH signaling. Overall, the meta-analysis revealed that the deletion of MCH signaling enhanced wakefulness, locomotor activity, aggression, and male sexual behavior and that MCH signaling deficiency suppressed non-REM sleep, anxiety, responses to novelty, startle responses, and conditioned place preferences. In contrast to the acute orexigenic effect of MCH, MCH signaling deficiency significantly increased food intake. Overall, the meta-analysis also revealed that the deletion of MCH signaling suppressed the body weight, fat mass, and plasma leptin, while MCH signaling deficiency increased the body temperature, oxygen consumption, heart rate, and mean arterial pressure. The lean phenotype of the MCH signaling-deficient mice was also confirmed in separate meta-analyses that were specific to sex and background strain (i.e., C57BL/6 and 129Sv). MCH signaling deficiency caused a weak anxiolytic effect as assessed with the elevated plus maze and the open field test but also caused a weak anxiogenic effect as assessed with the emergence test. MCH signaling-deficient mice also exhibited increased plasma corticosterone under non-stressed conditions, which suggests enhanced activity of the hypothalamic-pituitary-adrenal axis. To the best of our knowledge, the present work is the first study to systematically compare the effects of MCH signaling on behavioral and metabolic phenotypes.

## Introduction

Meta-analysis is a statistical method of combining the results of individual studies to obtain the most reliable conclusions [1]. In clinical medicine, the power of meta-analysis is indispensable for building feasible and practical guidelines for the treatment of diseases. Although the use of meta-analysis in basic biomedical research is rare, the demand has been increasing because many researchers are working on genetically modified mice to examine the roles of genes in animal behavior and metabolism, and the results of the behavioral studies are not always consistent across research laboratories [2]. Studies with positive results tend to be published in scientific journals with many readers, while those with negative results are easily overlooked because they are typically coupled with positive results and published in journals with fewer readers.

Melanin-concentrating hormone (MCH) is a 17-amino-acid cyclic neuropeptide that was originally isolated from teleost fish; in these fish, MCH causes the aggregation of melanosomes in the scales, which results in color changes [3]. MCH expression is restricted to the lateral hypothalamus of the brain, and MCH is not expressed outside of the brain. Analyses of the gene expressions in the hypothalamus of leptin-deficient mice have revealed the orexigenic effect of MCH in mammals [4]. There are one or two G-protein-coupled receptors for MCH: humans express MCHR1 and MCHR2, whereas rodents express only MCHR1 [5], [6]. In contrast to the restricted localization of MCH neurons in the lateral hypothalamic area and the zona incerta, MCH neurons send their projections broadly across the brain, and MCHR1 is also widely expressed in the brain [7]. The broad projections of MCH neurons suggest that MCH signaling may be involved in the regulation of a wide array of behaviors [6].

Consistent with the orexigenic effect of the injection of MCH into the cerebral ventricles [4], [8], [9], MCH-overexpressing mice exhibit increased food intake and develop mild obesity [10]. Conversely, MCH-deficient mice and MCH neuron-ablated mice exhibit decreased food intake and lean phenotypes [11], [12]. In addition to reduced food intake, increased energy expenditure also contributes to the lean phenotypes of MCH-deficient mice and MCH neuron-ablated mice [11]–[13]. Furthermore, MCHR1-deficient mice exhibit a lean phenotype and resistance to high-fat diet-induced obesity [14]–[17]. These results indicate that the MCH-MCHR1 system works to accumulate energy in the body; therefore, MCH-receptor antagonists are currently believed to be attractive therapeutic targets for the treatment of obesity. However, it should be noted that the lean phenotypes of the MCH- and MCHR1-deficient mice have not always been replicated [18], [19], and increased food intake by MCH-deficient mice has also been reported [13].

In addition to metabolic parameters, MCH signaling appears to be involved in a variety of behavioral phenotypes. MCH- and MCHR1-deficient mice exhibit decreased non-REM sleep time [20], [21], impaired olfactory function [22], reduced responses to novelty [19], suppressed startle responses [19], and reduced sucrose preference [23]. Moreover, MCH signaling-deficient mice exhibit increased aggression [22], alcohol preference [23], sexual motivation [22], and sociability [24]. However, the findings from behavioral analyses have not been consistent. For example, locomotor activity is one of the most commonly measured parameters, and the measurement of locomotor activity is highly automated; however, some groups have reported that MCH- or MCHR1-deficient mice exhibit increased locomotion [13], [15], [25], [26], while others have reported no differences [21], [22], [27], [28]. Inconsistent findings regarding the roles of MCH signaling in anxiety behavior have also been reported; one study reported anxiolytic phenotypes among MCHR1-deficient mice [24], whereas other groups have reported normal anxiety behavior in MCHR1-deficient mice [27], [29]. These results prompted us to perform a meta-analysis to examine the contributions of MCH signaling to behavioral and metabolic parameters.

We performed a meta-analysis of the previously reported data regarding MCH signaling-deficient mice, MCH-deficient mice, and MCHR1-deficient mice. Because genetic background and sex affect behavioral and metabolic characteristics, we also performed separate meta-analyses segregated by background strain and sex.

## Methods

### Search Strategy

**Figure 1:**
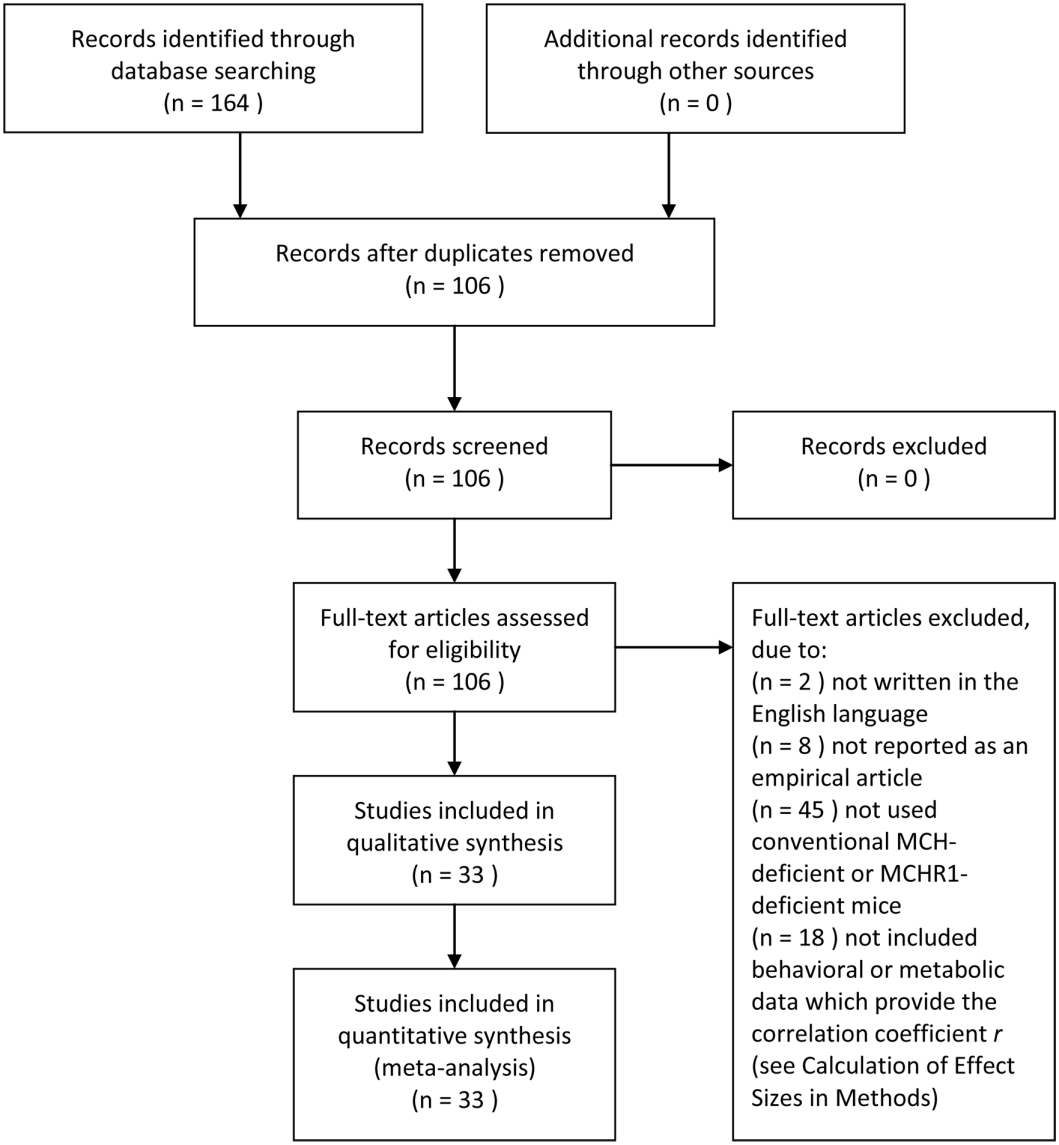
Flow chart showing the selection process of articles and the number in each period.

We performed an extensive electronic search to identify studies that had examined the effects of the deletion of MCH signaling on the behavioral phenotypes of mice. We searched PubMed (1948-Jan 2014) using the free-text search terms “melanin-concentrating hormone” or “melanin-concentrating hormone receptor” plus “knockout” or “deficient” on Jan 1, 2014. The first report about MCH signaling-deficient mouse was published in 1998 for MCH-deficient mouse [11], followed by the literature published in 2002 which was the first report about MCHR1-deficient mouse [30]. Additional searches were performed using the first, last, and corresponding authors of any of the reports identified in the initial PubMed search. The search was restricted to papers written in English because paper translations were not feasible within the scope of this review. No other restrictions were placed on the search criteria. This search retrieved 164 studies. The reference lists of the included papers and recent review papers in the field were hand-searched along with back issues to identify additional references (See PRISMA flowchart; Figure. 1).

### Study Selection

**Figure 2:**
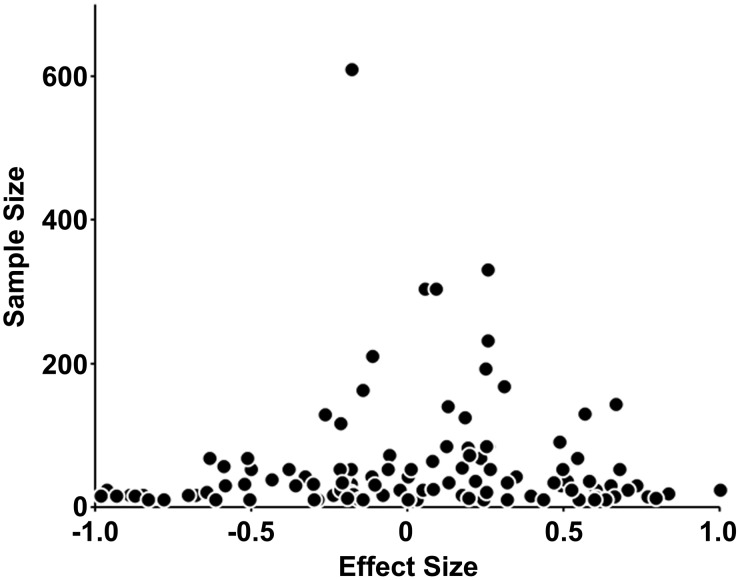
Funnel plot of effect sizes against sample sizes in overall meta-analysis. Black circles indicate behavioral or metabolic parameters.

A two-step procedure was used to identify the articles that were eligible for inclusion. First, the articles were assessed by reviewing their titles and abstracts to determine whether the articles met the following inclusion criteria: written in the English language; reported as an empirical article; used MCH-deficient or MCHR1-deficient mice; and included behavioral or metabolic experiments. These inclusion criteria were applied by two independent assessors, and the title and abstract reviews were conducted by same assessors. In cases in which the abstract provided insufficient information to make a final decision, the study was selected for full-text review. The full-text review was also conducted by two assessors. Next, the methodologies of the selected articles that were related to study quality were critically examined in full-text reviews. Studies that did not use wild-type littermates or wild-type animals from the same colony as the control mice were excluded. The parameter we used to index food intake was not normalized by body weight. The confirmation of the methodology was conducted by two assessors. The final decisions regarding inclusion were made by all of the present authors. Ultimately, 405 experiments from 33 studies were retained for the analysis. We did not include energy expenditure data in this study due to the inconsistency of the reported data (Figure 2D and Table 1 [15]).

**Table 1:** Results of meta-analysis.

MCH Signaling Function	Behavioral/Physiological/Biochemical Parameters	Sample Size	Number of Studies	r	95% Confidence Interval		P value vs. 0
					Lower Limit	Upper Limit	
Anxiety- and Mood-Related Behavior							
	Elevated Plus Maze	232	11	0.26	0.12	0.38	<0.05
	Elevated Plus Maze (Habituated Environment)	84	4	0.26	0.03	0.46	<0.05
	Emergence Test	42	2	−0.33	−0.02	−0.58	<0.05
	Emergence Test (Habituated Environment)	42	4	−0.12	0.21	−0.42	N.S.
	Exploratory Behavior	84	4	0.13	−0.10	0.34	N.S.
	Forced Swim test	68	2	0.23	−0.01	0.45	N.S.
	Open Field Test	330	9	0.26	0.15	0.36	<0.05
	Open Field Test (Habituated Environment)	168	8	0.31	0.15	0.45	<0.05
	Response to Handling	84	4	0.12	−0.11	0.34	N.S.
	Response to Novelty	84	4	0.25	0.03	0.45	<0.05
	Response to Supine Restraint	42	2	0.00	−0.32	0.32	N.S.
	Startle Response	42	2	0.35	0.04	0.60	<0.05
	Stress−Induced Hyperthermia	52	2	0.68	0.49	0.81	<0.05
Body Weight and Energy Metabolism							
	Body Length	54	3	0.17	−0.12	0.44	N.S.
	Body Temperature	68	3	−0.64	−0.46	−0.76	<0.05
	Body Temperature (Dark Phase)	32	2	−0.18	0.20	−0.51	N.S.
	Body Temperature (Light Phase)	32	2	−0.52	−0.19	−0.75	<0.05
	Body Weight	192	11	0.25	0.10	0.39	<0.05
	Body Weight (High Fat Diet)	90	5	0.49	0.30	0.64	<0.05
	Fat Mass	143	11	0.67	0.55	0.76	<0.05
	Fat Mass (High Fat Diet)	30	3	0.73	0.47	0.88	<0.05
	Food Intake	129	7	−0.26	−0.08	−0.43	<0.05
	Food Intake (High Fat Diet)	116	7	−0.21	−0.02	−0.40	<0.05
	Lean Mass	82	5	0.19	−0.04	0.41	N.S.
	Lean Mass (High Fat Diet)	16	2	0.17	−0.42	0.66	N.S.
	Liver Weight	24	1	0.70	0.42	0.86	<0.05
	Oxygen Consumption	24	3	−0.96	−0.90	−0.99	<0.05
	Oxygen Consumption (High Fat Diet)	15	2	−0.98	−0.94	−1.00	<0.05
Cardiovascular System							
	Heart Rate	16	1	−0.89	−0.71	−0.96	<0.05
	Heart Rate (Dark Phase)	16	1	−0.86	−0.64	−0.95	<0.05
	Heart Rate (Light Phase)	16	1	−0.68	−0.28	−0.88	<0.05
	Mean Arterial Pressure	16	1	−0.85	−0.61	−0.95	<0.05
	Mean Arterial Pressure (Dark Phase)	16	1	−0.24	0.29	−0.66	N.S.
	Mean Arterial Pressure (Light Phase)	16	1	−0.08	0.43	−0.55	N.S.
Endocrinology and Lab Data							
	Blood Ethanol Kinetics	72	4	−0.06	0.19	−0.30	N.S.
	Corticosterone Level	52	3	−0.50	−0.25	−0.69	<0.05
	CRF Level in CE	10	1	0.03	−0.61	0.65	N.S.
	CRF Level in PVN	10	1	0.55	−0.13	0.88	N.S.
	Estrous Cycling	20	1	−0.18	0.28	−0.58	N.S.
	Free Fatty Acid Level	24	1	0.05	−0.36	0.44	N.S.
	Ghrelin Level	34	2	0.13	−0.23	0.46	N.S.
	Glucose Level	124	7	0.18	−0.01	0.36	N.S.
	Glucose Level (High Fat Diet)	64	6	0.08	−0.21	0.35	N.S.
	Insulin Level	140	8	0.13	−0.05	0.30	N.S.
	Insulin Level (High Fat Diet)	30	3	0.49	0.11	0.75	<0.05
	Leptin Level	130	8	0.57	0.42	0.68	<0.05
	Leptin Level (High Fat Diet)	30	3	0.65	0.33	0.83	<0.05
	Leptin Level (per gram Fat)	17	2	−0.17	0.39	−0.65	N.S.
	Leptin Level (per gram Fat) (High Fat Diet)	36	4	0.51	0.16	0.74	<0.05
	Liver Triglyceride Level	24	1	0.60	0.26	0.81	<0.05
	Liver Triglyceride Level (High Fat Diet)	14	1	0.77	0.40	0.92	<0.05
	T4 Level	66	3	0.54	0.33	0.70	<0.05
	Total Cholesterol Level	24	1	1.00	1.00	1.00	<0.05
	Triglyceride Level	72	3	0.20	−0.05	0.42	N.S.
Learning and Memory							
	Spatial Learning Function	42	2	−0.21	0.12	−0.49	N.S.
Locomotion							
	Locomotor Activity	609	10	−0.18	−0.10	−0.26	<0.05
	Locomotor Activity (High Fat Diet)	15	2	−0.93	−0.77	−0.98	<0.05
	Locomotor Activity (Dark Phase)	56	5	−0.59	−0.36	−0.75	<0.05
	Locomotor Activity (Dark Phase) (High Fat Diet)	15	2	−0.87	−0.60	−0.96	<0.05
	Locomotor Activity (Light Phase)	32	2	−0.30	0.07	−0.60	N.S.
Reward System							
	Alcohol Preference	30	2	−0.59	−0.26	−0.79	<0.05
	Conditioned Place Preference	14	1	0.66	0.20	0.88	<0.05
	Motor Activation by Psychostimulants	38	1	−0.44	−0.13	−0.66	<0.05
	Sensitization to Psychostimulants	31	1	−0.11	0.28	−0.46	N.S.
	Sucrose Preference	15	1	0.39	−0.15	0.75	N.S.
Sensorimotor Function							
	Motor Coordination	210	10	−0.11	0.03	−0.25	N.S.
	Olfaction	13	1	0.60	0.08	0.87	<0.05
Sleep-Wakefulness							
	Non-REM Sleep (24 h)	68	3	0.54	0.33	0.70	<0.05
	Non-REM Sleep (Dark Phase)	52	3	0.50	0.24	0.69	<0.05
	Non-REM Sleep (Light Phase)	52	3	0.26	−0.03	0.51	N.S.
	REM Sleep (24 h)	52	3	−0.07	0.23	−0.35	N.S.
	REM Sleep (Dark Phase)	52	3	0.01	−0.28	0.30	N.S.
	REM Sleep (Light phase)	52	3	−0.18	0.11	−0.45	N.S.
	Wakefulness (24 h)	68	4	−0.51	−0.30	−0.68	<0.05
	Wakefulness (Dark Phase)	52	3	−0.38	−0.10	−0.60	<0.05
	Wakefulness (Light Phase)	52	3	−0.22	0.08	−0.48	N.S.
Social Behavior							
	Resident-Intruder Test	16	1	−0.70	−0.32	−0.89	<0.05
	Male Mating Behavior	10	1	−0.83	−0.43	−0.96	<0.05
	Social Interaction Test	30	1	−0.36	0.00	−0.64	<0.05
Aminergic and Peptidergic Signaling							
	Agouti-Related Protein Level in Hypothalamus	12	1	0.19	−0.43	0.69	N.S.
	DA Level in NAc	25	1	0.08	−0.32	0.46	N.S.
	DAT Level in CPu	24	2	−0.21	0.24	−0.59	N.S.
	DAT Level in GP	10	1	0.00	−0.63	0.63	N.S.
	DAT Level in NAc	34	3	−0.21	0.18	−0.54	N.S.
	DAT Level in OT	10	1	0.00	−0.63	0.63	N.S.
	DAT Level in SN	10	1	−0.16	0.53	−0.72	N.S.
	DAT Level in VTA	10	1	−0.29	0.42	−0.78	N.S.
	D1R Level in CPu	24	2	−0.03	0.41	−0.45	N.S.
	D1R Level in GP	10	1	0.00	−0.63	0.63	N.S.
	D1R Level in NAc	34	3	0.47	0.11	0.71	<0.05
	D1R Level in OT	10	1	0.24	−0.46	0.76	N.S.
	D1R Level in SN	10	1	0.24	−0.46	0.76	N.S.
	D1R Level in VTA	10	1	0.63	0.00	0.90	<0.05
	D2R Level in CPu	24	2	0.52	0.12	0.78	<0.05
	D2R Level in GP	10	1	0.00	−0.63	0.63	N.S.
	D2R Level in NAc	34	3	0.32	−0.06	0.62	N.S.
	D2R Level in OT	10	1	0.63	0.00	0.90	<0.05
	D2R Level in SN	10	1	0.24	−0.46	0.76	N.S.
	D2R Level in VTA	10	1	0.24	−0.46	0.76	N.S.
	NE Level in NAc	25	1	0.08	−0.32	0.46	N.S.
	NET Level in CPu	10	1	−0.14	0.53	−0.71	N.S.
	NET Level in GP	10	1	−0.78	−0.30	−0.95	<0.05
	NET Level in NAc	20	2	−0.64	−0.24	−0.86	<0.05
	NET Level in OT	10	1	−0.30	0.41	−0.78	N.S.
	NET Level in SN	10	1	−0.62	0.02	−0.90	N.S.
	NET Level in VTA	10	1	−0.51	0.18	−0.86	N.S.
	Neuropeptide Y Level in Hypothalamus	12	1	0.19	−0.43	0.69	N.S.
	NR1 Subunit Level in HPC	36	3	0.58	0.28	0.78	<0.05
	NR2A Subunit Level in HPC	36	3	0.22	−0.16	0.53	N.S.
	NR2B Subunit Level in HPC	36	3	0.22	−0.16	0.53	N.S.
	Orexin Level in Hypothalamus	12	1	−0.19	0.43	−0.69	N.S.
	Pro-Opiomelanocortin Level in Hypothalamus	12	1	0.79	0.40	0.94	<0.05
	5HT Level in NAc	25	1	0.08	−0.32	0.46	N.S.
	5HT Level in PFC	18	1	0.83	0.60	0.94	<0.05
	5HTT Level in CPu	10	1	0.00	−0.63	0.63	N.S.
	5HTT Level in GP	10	1	0.32	−0.39	0.79	N.S.
	5HTT Level in NAc	20	2	0.25	−0.26	0.65	N.S.
	5HTT Level in OT	10	1	0.60	−0.05	0.89	N.S.
	5HTT Level in SN	10	1	0.43	−0.27	0.83	N.S.
	5HTT Level in VTA	10	1	0.00	−0.63	0.63	N.S.
Others							
	Litter Size	304	2	0.05	−0.06	0.17	N.S.
	Mean Pup Mass	304	2	0.09	−0.02	0.20	N.S.
	Pup Mortality	163	1	−0.14	0.01	−0.29	N.S.

r = mean effect size, P value was calculated by Z test. CE, central amygdaloid nucleus; CPu, caudate-putamen; CRF, corticotropin-releasing factor; D1R, dopamine D1 receptor; D2R, dopamine D2 receptor; DA, dopamine; DAT, dopamine transporter; GP, globus pallidus; HPC, hippocampus; 5HTT, 5HT transporter; NAc, nucleus accumbens; NE, norepinephrine; NET, norepinephrine transporter; OT, olfactory tubercle; PFC, prefrontal cortex; PVN, paraventricular nucleus; SN, substantia nigra; VTA, ventral tegmental area.

### Coding of the Variables

The effects of the deletion of MCH signaling on behavioral and metabolic phenotypes were derived by coding the performances in behavioral tests or the values that resulted from physiological and biochemical assays (Table S1). Regarding the behavioral phenotypes, the types of behavioral tests and the parameters of the behavioral tests were coded. Regarding the metabolic phenotypes, the units of the physiological and biochemical assays were coded. The behavioral and metabolic phenotypes were also coded for the background strain, sex, age, and sample size. Three background strains (C57BL/6, 129, and 129×C57BL/6) were coded, but the substrain, such as C57BL/6J, C57BL/6N, 129SvEv, and 129SvEvBrd, was not considered. In some cases, the sex or age was not described, and these cases were excluded from the separate meta-analyses.

### Sensitivity Analysis

To assess potential publication bias, the tendency that significant results are more likely to be published than negative results, funnel plots were first created for the overall meta-analysis and for each separate meta-analysis. Because larger study samples tend to provide better estimates of the true effect sizes, inverted funnel shapes should be observed when the effect sizes are plotted against the sample sizes. Further, the funnel plots were assessed for asymmetry using the Egger test [1], [31]. Asymmetry of funnel plots suggests the publication bias due to the under-representation in the negative tail of the plot. Differences were considered significant at *p*<0.05.

### Calculation of Effect Sizes

We used the correlation coefficient *r* to index effect sizes. The value of the test statistics resulting from two-tailed *t*-tests, one-way analyses of variance (two groups), chi-squared tests (2×2), and Wilcoxon-Mann-Whitney tests were converted to the correlation coefficient *r* [32]. In some cases, the value of the test statistic was not reported. In these cases, we calculated the value of the test statistic from the *p*-value and the sample size *n* or the *df* [33]. Furthermore, if only the sample means and standard errors of two groups were reported, we calculated a *t*-value from these values. The obtained *r* values indicate how well a particular parameter correlates with the deletion of MCH signaling; an *r* of ±1.0 indicates a perfect (positive/negative) correlation. Correlations are positive when the parameter is suppressed or decreased in the MCH signaling-deficient mice. To estimate the 95% confidence intervals, we transformed the *r* values into Fisher’s *z* values. Fisher’s *z* is normally distributed, and its variance is 1/(*n*-3). We then calculated the 95% confidence interval the Fisher’s *z* metric and converted the Fisher’s *z* back to *r*. Statistical analysis was performed using Microsoft Excel.

### Analysis of Effect Sizes

The mean effect size of each parameter was calculated. In our analyses, the number of experiments that we used to calculate the mean of each parameter was small. Hence, there was not enough power for a test of heterogeneity. We assume that there is one true effect size which is shared by all the included experiments and conducted the analyses using the fixed-effect model [34]. To calculate the means, we calculated the weighted means of the Fisher’s *z*-transformed correlations and converted the results back to the *r* metric. Then, the sample sizes were summed in those parameters. In the overall meta-analysis, significance testing was performed using the 95% confidence intervals. If the confidence interval did not contain zero, we judged the effect of the deletion of MCH signaling on that parameter to be statistically significant. In the separate meta-analyses, we adopted the parameters which were balanced in terms of MCH/MCHR1 KO, background strain, and sex. Further, the mean effect sizes of each group that were judged to be statistically significant were compared with each other using *z*-tests. Differences were considered significant at *p*<0.05. In the present analysis, the correlations were considered strong when the lower limit of the 95% confidence interval was >0.3.

## Results

### Publication Bias

To assess potential publication bias, the tendency that significant results are more likely to be published than negative results, we examined the distributions of the included parameters (Table 1) by funnel plot, showing inverted funnel shapes (Figure. 2). The Egger tests assessing asymmetry of plotted parameters revealed no significant differences (*p*>0.05).

In the separate meta-analyses of the MCH and MCHR1 strains, the background strain, and each gender, the distributions of the included behavioral and metabolic parameters (Tables 2–4) exhibited inverted funnel shapes (Figures. S1–3). The Egger tests revealed no significant differences (*p*>0.05), which suggests that the results of the separate meta-analyses (Tables 2–4) were relatively robust and that the effects of publication bias were small.

**Table 2:** Results of meta-analysis in MCH KO or MCHR1 KO mice.

Behavioral/Physiological/Biochemical Parameters	MCH KO					P value vs. MCHR1 KO	MCHR1 KO				
	Sample Size	r	95% Confidence Interval		P value vs. 0		Sample Size	r	95% Confidence Interval		P value vs. 0
			Lower Limit	Upper Limit					Lower Limit	Upper Limit	
Body Weight	68	0.21	−0.06	0.46	N.S.	-	124	0.26	0.08	0.43	<0.05
Body Weight (High Fat Diet)	23	0.69	0.36	0.87	<0.05	N.S.	20	0.54	0.12	0.79	<0.05
Fat Mass	76	0.67	0.51	0.79	<0.05	N.S.	44	0.68	0.46	0.82	<0.05
Food Intake	31	−0.17	0.21	−0.51	N.S.	-	46	0.01	−0.29	0.31	N.S.
Food Intake (High Fat Diet)	45	0.16	−0.16	0.45	N.S.	-	20	−0.04	0.41	−0.47	N.S.
Glucose Level	21	0.11	−0.33	0.52	N.S.	-	24	0.21	−0.21	0.56	N.S.
Insulin Level	26	0.07	−0.35	0.47	N.S.	-	48	0.30	0.01	0.54	<0.05
Lean Mass	38	0.20	−0.14	0.50	N.S.	-	16	0.44	−0.07	0.77	N.S.
Leptin Level	16	0.31	−0.29	0.74	N.S.	-	48	0.77	0.61	0.86	<0.05
Locomotor Activity	39	−0.21	0.12	−0.51	N.S.	-	76	−0.13	0.10	−0.35	N.S.
Locomotor Activity (Dark Phase)	16	−0.72	−0.29	−0.91	<0.05	N.S.	32	−0.40	−0.04	−0.67	<0.05
Non-REM Sleep (24 h)	16	0.82	0.54	0.93	<0.05	N.S.	32	0.61	0.31	0.80	<0.05
Non-REM Sleep (Dark Phase)	16	0.79	0.48	0.92	<0.05	-	16	0.49	−0.01	0.79	N.S.
Non-REM Sleep (Light Phase)	16	0.43	−0.09	0.76	N.S.	-	16	0.49	−0.01	0.79	N.S.
REM Sleep (24 h)	16	0.45	−0.06	0.77	N.S.	-	16	−0.17	0.36	−0.61	N.S.
REM Sleep (Dark Phase)	16	0.34	−0.19	0.71	N.S.	-	16	−0.17	0.36	−0.61	N.S.
REM Sleep (Light Phase)	16	0.24	−0.29	0.65	N.S.	-	16	−0.17	0.36	−0.61	N.S.
Wakefulness (24 h)	16	−0.84	−0.58	−0.94	<0.05	N.S.	32	−0.58	−0.27	−0.78	<0.05
Wakefulness (Dark Phase)	16	−0.78	−0.46	−0.92	<0.05	-	16	−0.17	0.36	−0.61	N.S.
Wakefulness (Light Phase)	16	−0.45	0.06	−0.77	N.S.	-	16	−0.49	0.01	−0.79	N.S.

r = mean effect size, P value was calculated by Z test.

**Table 3:** Results of meta-analysis in C57BL/6, 129, or 129×C57BL/6 mice.

Behavioral/Physiological/Biochemical Parameters	C57BL/6					P value vs. 129	P value vs. 129×C57BL/6	129					P value vs. 129×C57BL/6	129×C57BL/6				
	Sample Size	r	95% Confidence Interval		P value vs. 0			Sample Size	r	95% Confidence Interval		P value vs. 0		Sample Size	r	95% Confidence Interval		P value vs. 0
			Lower Limit	Upper Limit						Lower Limit	Upper Limit					Lower Limit	Upper Limit	
Body Length	20	0.43	−0.01	0.73	N.S.	-	-	22	−0.10	0.33	−0.50	N.S.	-	-	-	-	-	-
Body Temperature	38	−0.50	−0.20	−0.72	<0.05	-	N.S.	-	-	-	-	-	-	30	−0.75	−0.54	−0.88	<0.05
Body Weight	102	0.54	0.37	0.67	<0.05	N.S.	-	38	−0.49	−0.17	−0.71	<0.05	-	-	-	-	-	-
Body Weight (High Fat Diet)	23	0.69	0.36	0.87	<0.05	-	-	7	0.54	−0.36	0.92	N.S.	-	-	-	-	-	-
Corticosterone Level	24	0.14	−0.27	0.52	N.S.	-	-	-	-	-	-	-	-	28	−0.84	−0.66	−0.93	<0.05
Elevated Plus Maze	106	0.38	0.19	0.54	<0.05	-	N.S.	44	0.08	−0.24	0.38	N.S.	-	42	0.36	0.05	0.61	<0.05
Elevated Plus Maze (Habituated Environment)	40	0.57	0.30	0.75	<0.05	-	-	44	−0.08	0.24	−0.38	N.S.	-	-	-	-	-	-
Emergence Test	40	−0.58	−0.31	−0.76	<0.05	-	-	44	−0.14	0.17	−0.43	N.S.	-	-	-	-	-	-
Emergence Test (Habituated Environment)	40	−0.24	0.09	−0.52	N.S.	-	-	44	−0.10	0.21	−0.40	N.S.	-	-	-	-	-	-
Exploratory Behavior	40	0.19	−0.15	0.48	N.S.	-	-	44	0.07	−0.24	0.37	N.S.	-	-	-	-	-	-
Fat Mass	56	0.70	0.51	0.82	<0.05	N.S.	-	23	0.61	0.18	0.84	<0.05	-	-	-	-	-	-
Fat Mass (High Fat Diet)	23	0.71	0.40	0.88	<0.05	N.S.	-	7	0.79	0.10	0.97	<0.05	-	-	-	-	-	-
Food Intake	31	−0.17	0.21	−0.51	N.S.	-	-	8	−0.86	−0.39	−0.97	<0.05	-	-	-	-	-	-
Food Intake (High Fat Diet)	45	0.16	−0.16	0.45	N.S.	-	-	7	−0.66	0.18	−0.94	N.S.	-	-	-	-	-	-
Ghrelin Level	24	0.28	−0.14	0.61	N.S.	-	-	-	-	-	-	-	-	10	−0.33	0.38	−0.79	N.S.
Glucose Level	23	0.21	−0.25	0.60	N.S.	-	-	8	0.22	−0.58	0.80	N.S.	-	-	-	-	-	-
Glucose Level (High Fat Diet)	37	0.13	−0.24	0.46	N.S.	-	-	7	0.83	0.20	0.97	<0.05	-	-	-	-	-	-
Insulin Level	9	−0.30	0.45	−0.80	N.S.	-	-	8	−0.20	0.59	−0.79	N.S.	-	-	-	-	-	-
Insulin Level (High Fat Diet)	23	0.53	0.11	0.79	<0.05	-	-	7	0.31	−0.58	0.86	N.S.	-	-	-	-	-	-
Lean Mass	38	0.20	−0.14	0.50	N.S.	-	-	8	0.36	−0.46	0.85	N.S.	-	-	-	-	-	-
Lean Mass (High Fat Diet)	9	0.55	−0.18	0.89	N.S.	-	-	7	−0.46	0.45	−0.90	N.S.	-	-	-	-	-	-
Leptin Level	9	0.43	−0.33	0.85	N.S.	-	-	8	0.41	−0.41	0.87	N.S.	-	-	-	-	-	-
Leptin Level (High Fat Diet)	23	0.66	0.31	0.85	<0.05	-	-	7	0.60	−0.28	0.93	N.S.	-	-	-	-	-	-
Leptin Level (per gram fat)	9	−0.31	0.44	−0.81	N.S.	-	-	8	0.00	−0.70	0.70	N.S.	-	-	-	-	-	-
Leptin Level (per gram fat) (High Fat Diet)	9	0.48	−0.27	0.87	N.S.	-	-	7	0.66	−0.19	0.94	N.S.	-	-	-	-	-	-
Locomotor Activity	39	−0.21	0.12	−0.51	N.S.	-	-	8	−0.93	−0.66	−0.99	<0.05	-	-	-	-	-	-
Locomotor Activity (High Fat Diet)	8	−0.94	−0.70	−0.99	<0.05	N.S.	-	7	−0.92	−0.55	−0.99	<0.05	-	-	-	-	-	-
Locomotor Activity (Dark Phase)	16	−0.72	−0.29	−0.91	<0.05	N.S.	-	8	−0.91	−0.57	−0.98	<0.05	-	-	-	-	-	-
Locomotor Activity (Dark Phase) (High Fat Diet)	8	−0.89	−0.50	−0.98	<0.05	N.S.	-	7	−0.85	−0.27	−0.98	<0.05	-	-	-	-	-	-
Motor Coordination	100	−0.08	0.13	−0.28	N.S.	-	-	110	−0.15	0.05	−0.33	N.S.	-	-	-	-	-	-
Non-REM Sleep (24 h)	32	0.61	0.31	0.80	<0.05	-	-	20	0.05	−0.40	0.48	N.S.	-	-	-	-	-	-
Non-REM Sleep (Dark Phase)	16	0.49	−0.01	0.79	N.S.	-	-	20	0.14	−0.32	0.55	N.S.	-	-	-	-	-	-
Non-REM Sleep (Light Phase)	16	0.49	−0.01	0.79	N.S.	-	-	20	−0.08	0.38	−0.50	N.S.	-	-	-	-	-	-
Open Field Test	160	0.24	0.09	0.39	<0.05	-	N.S.	44	0.06	−0.25	0.36	N.S.	-	80	0.45	0.26	0.61	<0.05
Open Field Test (Habituated Environment)	80	0.46	0.26	0.63	<0.05	-	-	88	0.16	−0.07	0.36	N.S.	-	-	-	-	-	-
Oxygen Consumption	16	−0.95	−0.85	−0.99	<0.05	N.S.	-	8	−0.98	−0.88	−1.00	<0.05	-	-	-	-	-	-
Oxygen Consumption (High Fat Diet)	8	−0.97	−0.83	−0.99	<0.05	N.S.	-	7	−0.99	−0.95	−1.00	<0.05	-	-	-	-	-	-
Response to Handling	40	-0.08	0.25	−0.39	N.S.	-	-	44	0.29	−0.02	0.55	N.S.	-	-	-	-	-	-
Response to Novelty	40	0.26	−0.07	0.54	N.S.	-	-	44	0.24	−0.07	0.51	N.S.	-	-	-	-	-	-
Response to Supine Restraint	20	0.00	−0.44	0.44	N.S.	-	-	22	0.00	−0.42	0.42	N.S.	-	-	-	-	-	-
Spatial Learning Function	20	−0.16	0.31	−0.56	N.S.	-	-	22	−0.25	0.19	−0.61	N.S.	-	-	-	-	-	-
Startle Response	20	0.15	−0.31	0.56	N.S.	-	-	22	0.50	0.10	0.76	<0.05	-	-	-	-	-	-
Stress Induced Hyperthermia	22	0.46	0.05	0.74	<0.05	-	N.S.	-	-	-	-	-	-	30	0.78	0.59	0.89	<0.05
T4 Level	18	0.79	0.51	0.92	<0.05	-	N.S.	-	-	-	-	-	-	24	0.54	0.18	0.78	<0.05
Triglyceride Level	24	0.55	0.19	0.78	<0.05	-	-	-	-	-	-	-	-	24	−0.17	0.25	−0.54	N.S.
Wakefulness (24 h)	32	−0.58	−0.78	−0.27	<0.05	-	-	20	0.07	−0.38	0.50	N.S.	-	-	-	-	-	-
Wakefulness (Dark Phase)	16	−0.17	0.36	−0.61	N.S.	-	-	20	−0.09	0.37	−0.51	N.S.	-	-	-	-	-	-
Wakefulness (Light Phase)	16	−0.49	0.01	−0.79	N.S.	-	-	20	0.22	−0.25	0.60	N.S.	-	-	-	-	-	-

r = mean effect size, P value was calculated by Z test.

**Table 4:** Results of meta-analysis in male or female mice.

Behavioral/Physiological/Biochemical Parameters	Male					P value vs. Female	Female				
	Sample Size	r	95% Confidence Interval		P value vs. 0		Sample Size	r	95% Confidence Interval		P value vs. 0
			Lower Limit	Upper Limit					Lower Limit	Upper Limit	
Body Weight	102	0.54	0.37	0.67	<0.05	-	52	0.12	−0.17	0.39	N.S.
Fat Mass	87	0.72	0.58	0.81	<0.05	N.S.	33	0.54	0.23	0.76	<0.05
Food Intake	46	0.01	−0.29	0.31	N.S.	-	24	−0.47	−0.08	−0.74	<0.05
Food Intake (High Fat Diet)	22	−0.57	−0.19	−0.80	<0.05	N.S.	22	−0.51	−0.11	−0.77	<0.05
Forced Swim Test	34	0.12	−0.23	0.44	N.S.	-	34	0.34	0.00	0.61	<0.05
Glucose Level	47	0.14	−0.18	0.43	N.S.	-	69	0.21	−0.04	0.43	N.S.
Glucose Level (High Fat Diet)	10	−0.09	0.57	−0.68	N.S.	-	10	−0.52	0.16	−0.87	N.S.
Insulin Level	67	−0.03	0.23	−0.29	N.S.	-	65	0.31	0.05	0.52	<0.05
Lean Mass	38	0.20	−0.14	0.50	N.S.	-	20	−0.09	0.37	−0.51	N.S.
Leptin Level	67	0.55	0.33	0.70	<0.05	N.S.	55	0.60	0.39	0.76	<0.05
Leptin Level (per gram Fat) (High Fat Diet)	10	0.55	−0.12	0.88	N.S.	-	10	0.38	−0.32	0.82	N.S.
Open Field Test	80	0.45	0.26	0.61	<0.05	-	34	0.12	−0.23	0.44	N.S.
T4 Level	24	0.54	0.18	0.78	<0.05	-	24	0.25	−0.17	0.60	N.S.
Triglyceride Level	24	0.14	−0.27	0.52	N.S.	-	24	−0.17	0.25	−0.54	N.S.

r = mean effect size, P value was calculated by Z test.

### Overall Meta-analysis: Comparison of the Effects of MCH Signaling on Phenotypes

The effects of MCH signaling on behavioral and metabolic parameters are summarized in Table 1. Overall, the meta-analysis revealed that the deletion of MCH signaling suppressed non-REM sleep, anxiety, response to novelty, startle response, stress-induced hyperthermia, conditioned place preference, and olfaction (*p*<0.05) and that MCH signaling deficiency enhanced locomotor activity, wakefulness, alcohol preference, motor activation by psychostimulants, aggression, male sexual behavior, and social interaction (*p*<0.05).

Regarding the metabolic parameters, the deletion of MCH signaling suppressed body weight, fat mass, leptin level, liver triglyceride level, and total cholesterol level (*p*<0.05), and MCH signaling deficiency enhanced food intake, body temperature, oxygen consumption, heart rate, mean arterial pressure, corticosterone level, and norepinephrine transporter level in the globus pallidus and nucleus accumbens (*p*<0.05). Additionally, MCH-signaling deficiency decreased D_1_ receptor levels in the nucleus accumbens and the ventral tegmental area, D_2_ receptor levels in the caudate-putamen and olfactory tubercle, NR1 subunit levels in the hippocampal formation, and 5-HT levels in the prefrontal cortex (*p*<0.05).

Strong correlations (95% confidential interval lower limit>0.3) were observed among the parameters related to body-weight regulation, including oxygen consumption, body temperature, fat mass, heart rate, arterial pressure, plasma leptin, locomotor activity, and serum total cholesterol. Other parameters that exhibited strong correlations (95% confidence interval with a lower limit>0.3) were non-REM sleep time, wake time, stress-induced hyperthermia, aggression, male sexual behavior, 5-HT levels in the prefrontal cortex, and norepinephrine transporter levels in the globus pallidus.

We did not find any inconsistency in the effect size between the earlier literatures before 2002 and recent literatures published in 2005 and later. For example, glucose level of MCH-deficient mouse reported in 1998 [11], is consistent with that reported in 2005 [13].

### Separate Meta-analyses for MCH Deficiency or MCHR1 Deficiency

The different effects of MCH deficiency and MCHR1 deficiency on the behavioral and metabolic parameters are summarized in Table 2. Separate meta-analyses for the deficiencies of the ligand and receptor revealed that both MCH deficiency and MCHR1 deficiency suppressed the body weight when the mice were on a high-fat diet, the fat mass, and the non-REM sleep time, whereas both MCH-deficient mice and MCHR1-deficient mice exhibited increased wake times and locomotor activities during the dark phase (*p*<0.05). MCHR1 deficiency produced significant effects on body weight, plasma insulin, and plasma leptin, and the MCH-deficient mice did not exhibit any significant effects in these parameters.

### Separate Meta-analyses by Background Strain

Behavioral studies of MCH and MCHR1 deficiency were performed using mice of the C57BL/6 (B6), 129/Sv (129), and 129-B6 mixed (mixed) backgrounds. Because each strain has different behavioral and metabolic characteristics due to genetic variations, the phenotypes of the gene-modified mice may vary among the different strains. Nevertheless, the phenotypes to which alterations in MCH signaling mainly contribute are thought to be recognizable in MCH signal-deficient mice of different strains. The results of the separate meta-analyses of the effects of the MCH and MCHR1 deficiencies on the behavioral and metabolic parameters are summarized in Table 3. In both the 129 and B6 backgrounds, the MCH and MCHR1 deficiencies decreased fat mass and increased oxygen consumption and locomotor activity (*p*<0.05). Interestingly, significant increases in food intake and body weight in the MCH-deficient mice were found only in the 129 (*p*<0.05) and not in the B6 background. The deletion of MCH signaling increased body temperature in the B6 and mixed backgrounds (*p*<0.05) and suppressed stress-induced hyperthermia in the B6 and mixed backgrounds (*p*<0.05). Due to the small number of studies that used mixed-background mice, data regarding body weight, fat mass, oxygen consumption, locomotor activity, and food intake were not available.

Under a high-fat diet, the deficiency in MCH signaling reduced the fat mass and increased oxygen consumption in both the 129 and B6 backgrounds (*p*<0.05). The lack of MCH signaling exhibited a suppressive effect on glucose levels under a high-fat diet only in the 129 mice (*p*<0.05), and no significant effect was observed in the B6 mice. Strain-specific effects were also observed in the following parameters: anxiolytic effects, as assessed with an open field test and elevated plus maze test, were observed in the MCH-deficient mice of both the B6 and mixed backgrounds (*p*<0.05), whereas no significant effects were observed in the 129 background mice; MCH-signaling deficiency in the B6 background produced a significant increase in wake time and a significant decrease in non-REM sleep time, and MCH-signaling deficiency in the 129 background did not produce significant changes in sleep/wakefulness behaviors.

### Separate Meta-analyses by Sex

To examine the differential role of MCH signaling in males and females, we performed separate meta-analyses, which are summarized in Table 4. MCH or MCHR1 deficiency suppressed fat mass and plasma leptin and enhanced high-fat diet intake in both sexes (*p*<0.05). The deletion of MCH signaling in the male mice suppressed body weight, anxiety, and plasma T4 (*p*<0.05). The deletion of MCH signaling in the female mice suppressed depressive behavior and plasma insulin (*p*<0.05) and enhanced food intake (*p*<0.05).

## Discussion

### The Role of MCH Signaling in Feeding Behavior

Nearly two decades ago and long after the report of the expression of MCH in the mammalian lateral hypothalamus [35], Qu et al. reported the orexigenic effect of MCH [4]. Because the lateral hypothalamus is known to be a “feeding center” [36], the orexigenic effects of MCH received much attention that resulted in numerous reports that used MCH and MCH receptor antagonists [8], [37], [38]. Consistent with these pharmacological studies, the first report of MCH-deficient mice found a mild reduction in food intake [11]. However, later reports failed to reproduce this hypophagia in MCH signaling-deficient mice [13], [15].

Surprisingly, the present meta-analysis revealed that MCH-signaling deficiency caused mild but significant increases in food intake when the mice were given normal or high-fat diets. However, the separate analyses of the MCH-deficient mice and the MCHR1-deficient mice did not show such an effect. The combined meta-analysis of the ligand- and receptor-deficient mice had sufficient statistical power to detect mild hyperphagia in the MCH signaling-deficient mice. The increased food intake of the MCH signaling-deficient mice does not contradict the acute orexigenic effects of MCH that have been reported by independent researchers based on pharmacological experiments [4], [8], [37], [38]; rather, these effects suggest that the acute orexigenic effects of MCH may be cancelled by long-term body-weight homeostasis mechanisms. For example, the administration of orexin, another orexigenic peptide that is expressed in the lateral hypothalamus, results in increased food intake in acute experiments but not in chronic injection experiments [39], [40]. Moreover, the overexpression of orexin renders mice resistant to diet-induced obesity [41], which suggests that short-term feeding behaviors and long-term body-weight homeostasis are regulated by distinct mechanisms.

### MCH Signaling Regulates Body Weight Homeostasis

Our meta-analysis confirmed that the body weights were reduced and that oxygen consumption increased in the MCH signaling-deficient mice on both normal chow and high-fat diets. Thus, the lean phenotype of the MCH signaling-deficient mice is likely due to increased energy output and not due to reduced energy input. The increased food intake of the MCH signaling-deficient mice may be caused by feedback regulation that maintains stable body weights. Consistent with this notion, our meta-analysis revealed that the MCH signaling-deficient mice exhibited decreased plasma leptin levels; leptin is a major anorexigenic hormone that is secreted from adipose tissues. Suppressive effects of MCH signaling deficiency on the weight gains of mice on high-fat diets were also found in the separate meta-analyses of the MCH-deficient, MCHR1-deficient, B6, male, and female mice. The lack of a suppressive effect on diet-induced obesity in the 129 background may have resulted from the fact that the wild-type 129 strain is resistant to diet-induced obesity, which, due to a floor effect, would make it difficult to observe a suppressive effect on body-weight gain [13].

The major source of energy output in rodents is the brown adipose tissue; the thermogenic activity of this tissue is regulated by the sympathetic nervous system [42]. Consistently, MCH neurons provide polysynaptic projections to the sympathetic nervous system and the skeletal muscle [43]. The loss of MCH signaling results in enhanced sympathetic tone, which increases energy output. The increased heart rates and higher arterial pressures in the MCH signaling-deficient mice also support the presence of enhanced sympathetic tone. The stress-induced hyperthermia of the MCH signaling-deficient mice may be partly due to the enhanced activity of the sympathetic nervous-brown adipose tissue system [44]. The present meta-analysis also revealed increased locomotion in the MCH signaling-deficient mice on both normal chow and high-fat diets; this increased locomotion would also contribute to energy output via skeletal muscle activity.

### MCH Signaling Regulates Sleep/Wakefulness Behavior

MCH neurons are actively firing during non-REM sleep and are more active during REM sleep. Our meta-analysis confirmed that MCH signaling deficiency enhanced wakefulness, suppressed non-REM sleep, and did not alter REM sleep. Because MCH neurons project to the locus coeruleus [7], which is crucial for wakefulness [45], [46], the loss of the inhibitory input of the MCH neurons onto the locus coeruleus may result in enhanced wakefulness.

The lack of a significant effect on REM sleep seems to be inconsistent with the fact that the highest activity levels of the MCH neurons occur during REM sleep [47]. However, the optogenetic activation of MCH neurons in MCHR1-deficient mice enhances REM sleep [48], which suggests that the MCH peptide released from the MCH neurons is not crucial for the generation of REM sleep.

### MCH Signaling Regulates Anxiety and Stress Responses

There have been contradictory reports about the anxiety-related behavior of MCH signaling-deficient mice [19], [24], [27], [49], [50]. Our meta-analysis did not produce clear conclusions regarding the effects of MCH signaling deficiency on anxiety; weak anxiolytic effects were found for in the elevated plus maze and open field tests, but weak anxiogenic effects were found in the emergence test. The increased plasma corticosterone levels during non-stressed conditions observed in the MCH signaling-deficient mice suggest that the activity of the hypothalamic-pituitary-adrenal (HPA) axis is enhanced and that these mice should tend to exhibit anxiogenic behavior [51]. Consistent with the enhanced activity of the HPA axis, the MCH signaling-deficient mice exhibited increased stress-induced hyperthermia, startle responses, and responses to novelty.

### The Role of MCH Signaling in Reward-seeking Behavior and Addiction

MCHR1 is most strongly expressed in the nucleus accumbens [7], which, along with the midbrain dopamine neurons, constitutes the neural circuitry that regulates reward-seeking behavior and addiction. Consistently, our meta-analysis revealed that the MCH signaling-deficient mice exhibited decreased cocaine-induced conditioned place preferences, which suggests that the MCH signaling-deficient mice are resistant to drug addiction. The increased locomotion of the MCH-deficient mice may have resulted from alterations in the mesolimbic dopamine system. Alterations in the dopaminergic system may also be involved in the hyperphagia exhibited by the MCH signaling-deficient mice. Although MCH signaling-deficient mice exhibit increased alcohol intake and a seemingly contradictory resistance to psychostimulants, the molecular target of alcohol in the brain is not the dopamine transporter but the GABA A receptor [52]. Thus, the neural substrates of alcohol-seeking behavior are thought to be distinct from those related to addictive behavior associated with dopamine-related psychostimulants.

### MCH Signaling Regulates Aggression and Sexual Motivation

The present meta-analysis revealed that MCH-signaling deficiency was strongly correlated with enhanced aggression and male sexual behavior (*r* = −0.704 and *r* = −0.832, respectively). MCHR1 is expressed in the ventromedial nucleus of the hypothalamus [7], which contains neurons that are involved in attack behavior and male sexual behavior [53], [54]. MCHR1 expression in the medial amygdala also plays an important role in aggression [55]. Defects in the olfactory system of MCH-deficient mice might be involved in the alterations of aggression and male sexual behavior [22].

### Therapeutic Implications

Based on an anorexic effect of the MCH peptide and the lean phenotypes of the MCH-deficient and MCHR1-deficient mice, MCHR1 antagonists have been recognized as attractive targets for the treatment of obesity. Continued efforts to develop small compounds that antagonize MCH signaling have extended the list of MCHR1 antagonists; some of these antagonists have been examined in clinical trials [37], [38], [56]–[58]. The current meta-analysis suggests that in addition to the treatment of obesity, possible therapeutic targets of MCHR1 antagonists include anxiety disorder, depressive disorder, hypersomnia, sleepiness, male sexual dysfunction, drug addiction, and hypotension. The possible side effects of the clinical use of MCHR1 antagonists include insomnia, hypersexuality, aggression, and hypertension.

The prominent difference in MCH signaling between humans and rodents is that humans express MCHR2. Although MCHR2 is expressed in the brain in a pattern similar to that of MCHR1, the downstream target of MCHR2 is different from that of MCHR1 because MCHR2 is a Gq-coupled receptor, whereas MCHR1 is a Gi-coupled receptor [59]–[61]. Thus, it is difficult to predict the clinical effects of MCHR1 antagonists in humans without an understanding of the physiological role of MCHR2 signaling. Indeed, a phase I clinical trial of an MCHR1 antagonist was discontinued due to the vivid dreams experienced by the subjects during the first week of treatment (NGD-4715; http://phx.corporate-ir.net/phoenix.zhtml?c=). This result could not be predicted based on the suppression of REM sleep following the injection of MCHR1 antagonists or the normal REM sleep of the MCH signaling-deficient mice.

### Limitations and Strengths

Studies that utilized mice in which the MCH neurons were ablated with ataxin-3 or diphtheria toxin were excluded from our meta-analysis because the lack of the GABAergic neurotransmission of the MCH neurons may result in behavioral alterations that are independent of the MCH-MCHR1 signal. Nevertheless, MCH neuron-ablated mice are lean and exhibit increased oxygen consumption, hyperlocomotion, and reduced responses to cocaine; these phenotypes are consistent with the results of the current meta-analysis of MCH signaling-deficient mice [12], [62].

The present meta-analysis is not free of limitations. The sample sizes varied across the parameters: many of the included studies examined body weight and food intake, but few studies examined oxygen consumption, largely because a special experimental apparatus is required to measure and control oxygen and carbon dioxide levels to allow for indirect calorimetry. The numbers of studies that we used to calculate the means of each parameter were small. Hence, there was not enough power for a test of heterogeneity. Furthermore, the number of parameters used for separate meta-analysis was small because we adopted the parameters which were balanced in terms of genotypes, sex, and strains.

To the best of our knowledge, the present work is the first study to systematically compare the effects of MCH signaling on behavioral and metabolic phenotypes. Our analyses revealed the robust contribution of MCH signaling to energy metabolism, reward behavior, and anxiety. Large numbers of researchers and international consortia are working on phenotyping genetically modified mice [63], and in combination with the remarkable progress that has been made in genome-editing techniques, this work has rendered the production of genetically modified mice easier and faster. Thus, the amounts of behavioral and metabolic data related to genetically modified mice will continue to increase. To obtain relevant conclusions from these large datasets, meta-analyses will become increasingly important in basic biomedical research.

## Supporting Information

Figure S1 **Funnel plot of effect sizes against sample sizes in separate meta-analysis by ligand-receptor.** Black circles indicate behavioral or metabolic parameters in MCH KO (left panel) or MCHR1 KO mouse (right panel).
(TIF)

Figure S2 **Funnel plot of effect sizes against sample sizes in separate meta-analysis by background strain.** Black circles indicate behavioral or metabolic parameters in C57BL/6 (left panel), 129 (middle panel), or 129×C57BL/6 mouse (right panel).
(TIF)

Figure S3 **Funnel plot of effect sizes against sample sizes in separate meta-analysis by sex.** Black circles indicate behavioral or metabolic parameters in male (left panel) or female mouse (right panel).
(TIF)

Table S1 **A descriptive summary of the studies used in the meta-analysis.**
(XLSX)

Checklist S1 PRISMA Checklist.
(DOC)
