# Supplementary figures and images for: Meta-Analysis of Melanin-Concentrating Hormone Signaling-Deficient Mice on Behavioral and Metabolic Phenotypes
Source: PLoS One. 2014 Jun 12;9(6):e99961. doi: 10.1371/journal.pone.0099961 (PMC4055708; doi:10.1371/journal.pone.0099961)

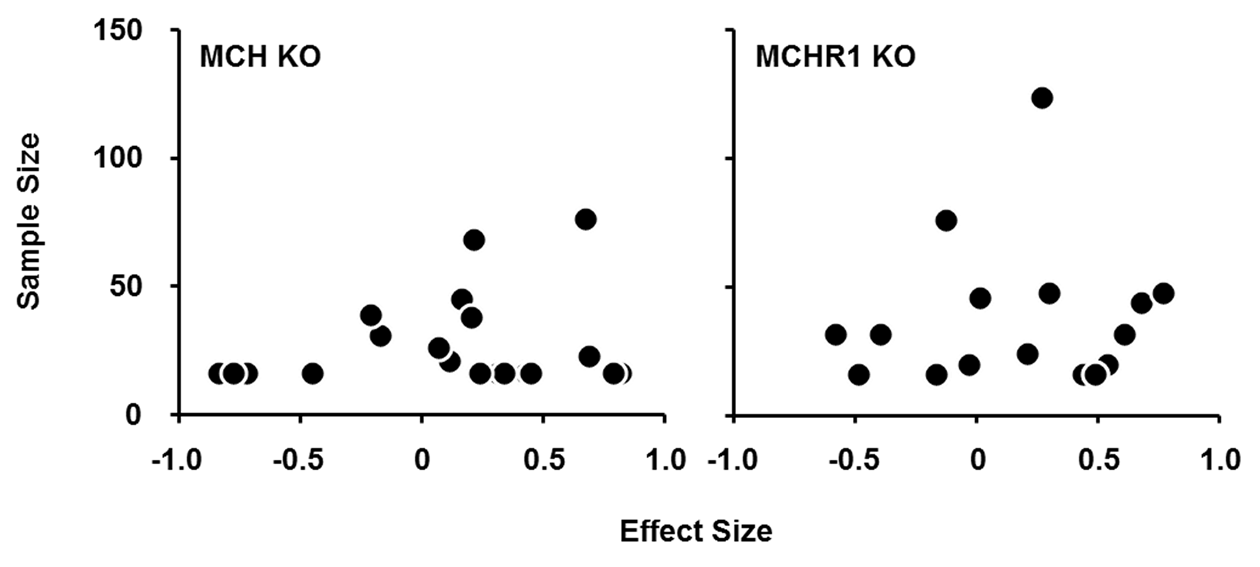

Supplement: Figure S1 — Funnel plot of effect sizes against sample sizes in separate meta-analysis by ligand-receptor. Black circles indicate behavioral or metabolic parameters in MCH KO (left panel) or MCHR1 KO mouse (right panel). (TIF) [file pone.0099961.s001.tif]

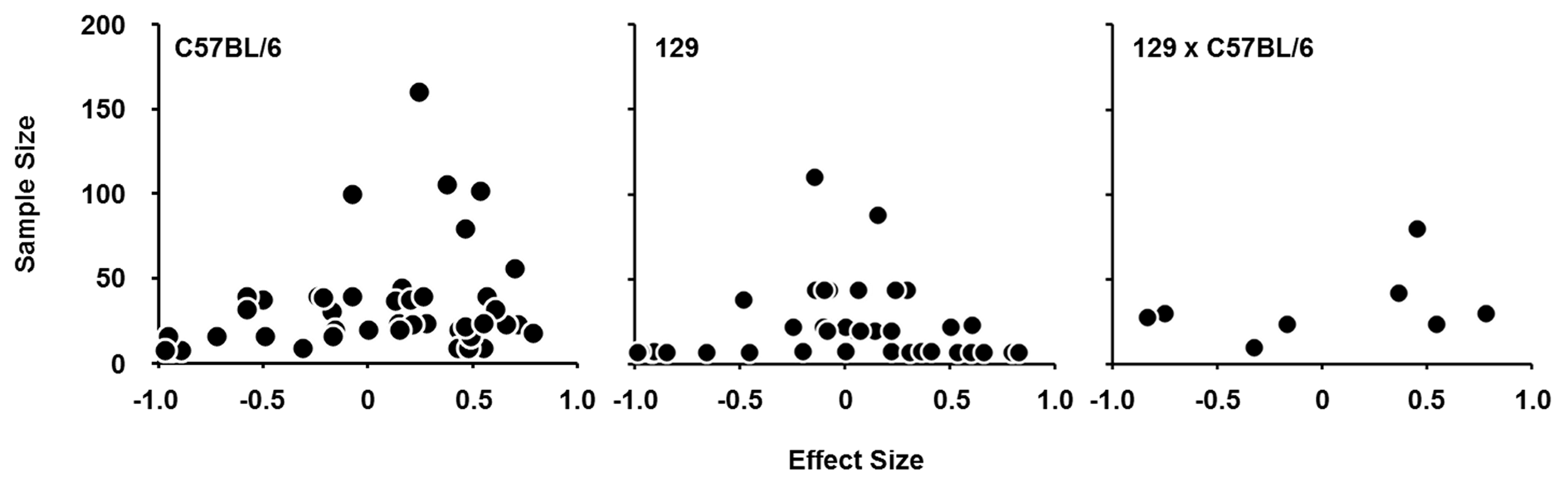

Supplement: Figure S2 — Funnel plot of effect sizes against sample sizes in separate meta-analysis by background strain. Black circles indicate behavioral or metabolic parameters in C57BL/6 (left panel), 129 (middle panel), or 129×C57BL/6 mouse (right panel). (TIF) [file pone.0099961.s002.tif]

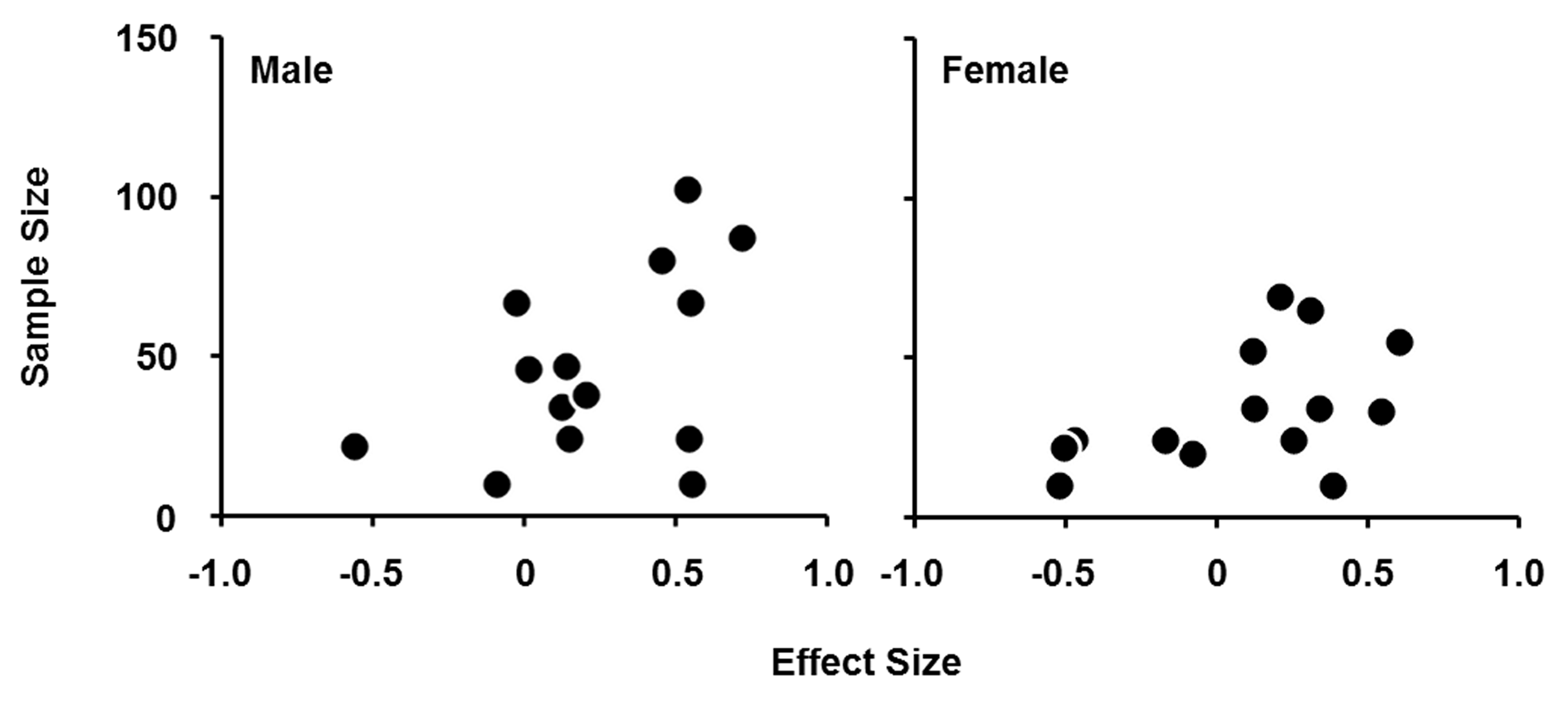

Supplement: Figure S3 — Funnel plot of effect sizes against sample sizes in separate meta-analysis by sex. Black circles indicate behavioral or metabolic parameters in male (left panel) or female mouse (right panel). (TIF) [file pone.0099961.s003.tif]
